# Isoprene Emissions from Downy Oak under Water Limitation during an Entire Growing Season: What Cost for Growth?

**DOI:** 10.1371/journal.pone.0112418

**Published:** 2014-11-10

**Authors:** Anne-Cyrielle Genard-Zielinski, Elena Ormeño, Christophe Boissard, Catherine Fernandez

**Affiliations:** 1 Institut Méditerranéen de Biodiversité et d’Ecologie marine et continentale (IMBE) Aix Marseille Université, CNRS, IRD, Avignon Université, Technopôle Arbois-Méditerranée, France; 2 Laboratoire des Sciences du Climat et de l’Environnement (LSCE-IPSL), Unité Mixte CEA-CNRS-UVSQ (Commissariat à l’Energie Atomique, Centre National de la Recherche Scientifique, Université de Versailles Saint-Quentin-en-Yvelines), Gif-sur-Yvette, France; Centro de Investigación y de Estudios Avanzados, Mexico

## Abstract

Increases in the production of terpene- and phenolic-like compounds in plant species under abiotic stress conditions have been interpreted in physiological studies as a supplementary defense system due to their capacity to limit cell oxidation. From an ecological perspective however, these increases are only expected to confer competitive advantages if they do not imply a significant cost for the plant, that is, growth reduction. We investigated shifts of isoprene emissions, and to a lesser extent phenolic compound concentration, of *Quercus pubescens* Willd. from early leaf development to leaf senescence under optimal watering (control: *C*), mild and severe water stress (*MS, SS*). The impact of water stress was concomitantly assessed on plant physiological (chlorophyll fluorescence, stomatal conductance, net photosynthesis, water potential) functional (relative leaf water content, leaf mass per area ratio) and growth (aerial and root biomass) traits. Growth changes allowed to estimate the eventual costs related to the production of isoprene and phenolics. The total phenolic content was not modified under water stress whereas isoprene emissions were promoted under *MS* over the entire growing cycle despite the decline of *P_n_* by 35%. Under *SS*, isoprene emissions remained similar to *C* all over the study despite the decline of *Pn* by 47% and were thereby clearly uncoupled to *Pn* leading to an overestimation of the isoprene emission factor by 44%. Under *SS*, maintenance of isoprene emissions and phenolic compound concentration resulted in very significant costs for the plants as growth rates were very significantly reduced. Under *MS,* increases of isoprene emission and maintenance of phenolic compound concentration resulted in moderate growth reduction. Hence, it is likely that investment in isoprene emissions confers *Q. pubescens* an important competitive advantage during moderate but not severe periods of water scarcity. Consequences of this response for air quality in North Mediterranean areas are also discussed.

## Introduction

Water limitation induced by climate change is going to affect particularly the Mediterranean area. Global models show, for the next century, a rise of 4°C [1] and a reduction of 30% of precipitation during summer [2]. In this region, with typical long dry and hot summers, vegetation undergoes an accentuated seasonal water stress. It has been shown that secondary metabolites both, volatile (Biogenic Volatile Organic Compounds or BVOC) and non-volatile (phenolic compounds) could help plants to overcome climate-related stress conditions, due to the protection they confer against thermal stress and [3],[4], overall, against any environmental constraint leading to cell oxidation (water stress, air pollution) [5]–[7]. Physiological studies indicate that phenolic compounds and isoprene cooperate to scavenge reactive oxygen species, thereby resulting in limited cell oxidation [4],[6],[8],[9]. It has been suggested that isoprene primarily participates to the enhancement of phospholipidic interactions between thylakoid membranes which are damaged under environmental constraints, thereby reducing cell oxydation [9]. However, from an ecological perspective, the physiological action of these secondary metabolites is not enough to conclude that increases in leaf phenolic content or isoprene emission rates confer competitive advantages for plants coping environmental stresses. This can be concluded if plant resource allocation for secondary metabolite production does not imply a cost for the plant in terms of growth reduction.

Isoprene (2-methyl, 1–3 butadiene, C_5_H_8_) represents 50% of total BVOC emissions at the global scale [10]. This volatile secondary metabolite impacts air pollutant formation including tropospheric ozone and Secondary Organic Aerosol (SOA) [11],[12]. These processes are particularly important in the Mediterranean region where ozone thresholds are exceeded numerous times every year, especially during summer, and particulate pollution has become a major concern for air quality.

Isoprene biosynthesis is dependent on photosynthesis and enzymatic processes and is therefore mainly controlled by light and temperature [13]–[16]. By contrast, the impact of drought on isoprene emission is controversial as reviewed by Peñuelas and Staudt (2010) [17], despite the well-known negative impact of drought on photosynthesis. These authors showed that around 50% of the studies relating isoprenoid emissions and drought found an emission decrease while 25% found an increase and the remaining reported no change, such variability being due to water stress intensity, timing and species.

The common point of most previous investigations focused on isoprene emission response to water limitation is that water stress is applied by stopping water supply for days or weeks (not an entire growing season) until a very significant decline of water potential, photosynthetic activity or stomatal conductivity is observed [18]–[24]. Under mild water stress, during the first days of water withholding, isoprene has been found to be slightly stimulated [22],[23] or unchanged [19],[24],[25] despite a decline of CO_2_ assimilation. Thereafter, when water availability becomes highly scarce, isoprene decline continues for a length of time which depends on the species studied, before emissions are fully inhibited [18]–[20]. Since isoprene emission occurs even when water stress dramatically inhibits photosynthesis due to stomata closure, emissions are uncoupled from photosynthesis during induced water stress. Funk et al. (2004) [23] demonstrated that in water-stressed plants isoprene production was not supported by recently assimilated carbon (as occurred in non-stressed plants), but rather by alternative carbon sources. These results suggest, on the one hand, that temperature and light dependency of isoprene emissions are weakened under this type of abiotic constraint, and, on the second hand, that isoprene confers resistance against water stress.

The objectifs of the present study were to **(i)** test the response of *Quercus pubescens* Willd. - a deciduous drought-resistant species [26] - against mild and severe water stress in terms of secondary metabolism (carbon-based secondary metabolites) with concomitant analysis of growth traits (using above and below–ground biomass as a proxy), physiological traits (photosyntheis, stomatal conductance, chlorophyll fluorescence, stem water potential) and functional traits (leaf mass to area ratio, leaf relative water content). We focused on isoprene emissions as a carbon-based secondary metabolite and, to a lesser extent, on phenolic compound concentrations, since *Q. pubescens* accounts for the major isoprene emitter in Northern Mediterranean regions (10–50 nmol.m^−2^.s^−1^) [18],[20],[27]; **(ii)** assess whether plant investment in isoprene emissions under water stress implies a cost in terms of growth reduction and requires an important fraction of the recently assimilated CO_2_; **(iii)** test the adequacy of the Guenther et al., (1993) (G93) algorithm [13] to calculate isoprene emission factors according to light and temperature under the different watering treatments. To attend such objectifs we performed a long-term study under greenhouse conditions consisting of three watering treaments (optimal watering, mild and severe water withholding) applied on *Q. pubescens* saplings over an entire growing season. We hypothesize that mild water stress promotes the production of carbon-based secondary metabolites based on their antioxidant properties and hence, the benefits they confer to plants under moderate stress conditions [6] and previous findings that report a rise. Our hypothesis is also based on the Growth Differentiation Balance Hypothesis (GDBH) [28] which states that moderate resource availability impacts growth rather than photosynthesis, resulting in an increasing carbon pool available to synthesize secondary metabolites. For severe stress however, we expect a decline of isoprene emissions based on both, previous findings performed over days or weeks of full watering withholding and the GDBH. The theory postulates that both growth and photosynthesis are constrained and both baseline maintenance and primary metabolic processes receive priority for carbon allocation compared to carbon-based secondary metabolites. We also hypothesize that, under water scarcity, estimations of isoprene emissions using the G93 algorithm are significantly different from those experimentally obtained considering the uncoupling between isoprene emissions and photosynthesis, and so with light [18]–[20],[29].

## Materials And Methods

### Ethic Statement

The experiment took place under greenhouse conditions in a national nursery in Aix-en-Provence (5°24′31.5″E, 43°30′35.8″N Southern France). The director of this nursery (Patrice Brahic) gave permission to conduct the study on this site. No specific permissions were required for these locations/activities, and studies did not involve endangered or protected species.

### Experimental Design

Four year-old *Q. pubescens* saplings were grown into 6 L pots, containing natural soil from a national experimental Downy oak forest (https://o3hp.obs-hp.fr/index.php/fr/). Greenhouses were built with transparent polycarbonate plates (Ombrex) which allowed ∼90% of the photosynthetic photon flux density (PPDF) to pass through. Environmental greenhouse conditions (air temperature and relative humidity) were recorded with HOBO devices (U12-012 HOBO data logger, temp/RH/light/ext channel).

Water withholding started in April, one week before measurements took place, and stopped late November, during leaf senescence. Saplings were divided into three groups corresponding to three watering treatments: (i) control *(C)*, where saplings were watered to reach and maintain soil field capacity, (ii) mild stress *(MS)*, and (iii) severe stress *(SS)* where saplings received 20% and 10% of soil field capacity respectively. Below 10% of soil field capacity, plants reached their wilting point. The degree of water stress under *MS* and *SS* was monitored by regularly checking sapling physiological activity. As net photosynthesis (*P_n_*) was below 1 µmol_CO2 _m^−2 ^h^−1^, saplings were punctually rewatered (this was only necessary during one day under *SS* just before June measurements, and during another day under *MS* just before July measurements), allowing to follow *Q. pubescens* response to water withholding all over an entire growing season (8 months). A non-destructive set of 15 saplings (5 *C*, 5 *MS* and 5 *SS*) was studied from April to November for gas exchange, isoprene emission and chlorophyll fluorescence measurements. Growth was also studied on this set of plants at the end of the study, at leaf senescence (i.e. in November). A destructive set of 15 different saplings (5 per treatment) was also used each month to measure soil water status, stem water potential and phenolic compound concentration. A total of 105 pots were thus used.

### Measurement Of Water Status In Soil And Plants And Leaf Mass Per Area Ratio

All parameters described herein were studied in the destructive set of saplings over the entire growing season and, only at the end of the study (November), they were also studied on the non-destructive set of saplings. Soil relative water content (SRWC) was measured using 100 g of soil harvested from each pot. Plant water status was estimated through two parameters: midday stem water potential (*ψ*_mid_) - measured using a pressure chamber (PMS instrument, Co. Oregon USA) - and leaf relative water content (LRWC) calculated following the formula: LRWC =  (FM-DM)/(SM-DM) where FM is fresh mass, DM is dry mass and SM is saturated mass. Leaf dry mass was measured by weighing the leaves after they had reached constant mass in an oven at 70°C. Leaf mass to area ratio (LMA), an indicator of leaf sclerophylly [30], was calculated by dividing leaf dry mass by leaf area, which was calculated using a leaf area meter (AM300 Bioscientific).

### Chlorophyll Fluorescence

Fluorescence was measured on the non-destructive set of plants after isoprene emission collection was performed. Fluorescence of three fully expanded leaves of each sapling was studied and results were averaged. Measurements were performed using a portable Hansatech Fluorescence Monitoring System (FMS 2) allowing computer-assisted data acquisition. The variable to maximum fluorescence ratio (*Fv/Fm*) was measured in the non-energized state after darkness adaptation of about 30 min using a leaf clip holder for adapting leaves to darkness. *Fv/Fm* is a reliable indicator of the maximal potential efficiency of excitation capture by open PSII in dark-adapted conditions, was also measured to estimate the functional state of the photosynthetic apparatus under the three watering treatments. The apparent maximum photosynthetic electron transport (*ETR_max_*) leading to carbon fixation was measured from light-adapted leaves as shown in Ormeño et al. (2009) [31].

### Leaf Gas Exchange

Net photosynthesis (*P_n_*) and stomatal conductance to water vapour (*G_w_*) were measured on the same saplings all over the experimentation, i.e. on the non-destructive set of saplings. The same leaf of each sapling was always studied over the growing season in order to eliminate leaf-to-leaf variability. *P_n_* and *G_w_* were measured using a 6 cm^2^ portable leaf LED cuvette (6400-02B LED Light Source Light-Emitting Diode) for PPFD control, coupled to an InfraRed Gas Analyzer (Li-cor 6400 XT system, Li-cor Lincoln, Nebraska, USA). Upstream, flow rate entering the cuvette monitored by the Li-cor 6400 XT device, was fixed at 1 l.min^−1^. Downstream, flow rate was set at 100 mL.min^−1^ using a mass flow controller (Bronckhorst, F-201CV-1K0-AAD-22-V) connected to a pump (E99335; Fisher Scientific). Most leaf gas exchange measurements were studied under standard conditions for isoprene emission (i.e. 30°C temperature and 1000 µmol m^−2 ^s^−1^ PPFD) as described by Guenther *et al.* (1993) [14]. Additionally, in May, June and July leaf gas exchanges of some saplings (n = 9, n = 3, n = 8 respectively) were not only measured under standard conditions but also under natural conditions of temperature and PPFD which ranged between 18 and 39°C and 384 and 1800 µmol.m^−2^.s^−1^ respectively.

### Isoprene Sampling And Analysis

The leaf cuvette previously described was modified in order to sample isoprene emissions from the non-destructive set of plants. Air exiting the cuvette was collected into glass tubes (15 cm long and 3 mm inner diameter) containing 60 mg tenax TA and 140 mg carbotrap. Glass tubes were attached to the cuvette using FPE tubing. For each sapling, isoprene collection took place simultaneously using two different glass tubes for 15 min at 100 mL.min^−1^ using a mass flow controller and a pump. Isoprene emissions collected onto both tubes were averaged. As it was previously described in the “leaf gas exchanges” section, isoprene emissions were mostly measured under standard conditions all over the experimentation excepting May, June and July, where isoprene emissions of some saplings were additionally collected under natural conditions of temperature and light. These additional samplings allowed to compare the experimentally measured isoprene emission factors (i.e. at 30±1°C and 1000±50 µmol m^−2 ^s^−1^ of PPFD) to the theoretical isoprene emission factor obtained using the G93 algorithm under the three watering treatments.

Isoprene was analysed with a gas chromatography (HP 6890N), coupled to an injector for thermal desorption (Gerstel TDS3/CIS4) and a mass spectrometer (HP 5973). A capillary column (Al/KCl) was used (30 m long, 250 microns of internal diameter and 5 microns of film thickness). The carrier gas was helium maintained at a constant flow of 1 ml min^−1.^ The desorption program was: pre-cooling to −50°C, 10 min-desorption at 250°C to 50 mL min^−1^, and then 3 min during injection maintained at 250°C. The analysis program was: 40°C to 200°C at 20°C min^−1^, maintained for 1 min at 200°C. The transfer line temperature was 200°C. The mass spectrometer fragmentation was done by electron impact at 70 eV, the source temperature was 230°C, the quadrupole temperature was set at 250°C and the potential difference of the electron multiplier was 1400. Data acquisition was done in scan mode from 40 to 150 amu.

### Concentration Of Phenolic Compounds

Total water-soluble phenolics of the destructive set of saplings (and in November for the non-destructive set of saplings) were extracted based on the method described by Singleton and Rossi (1965) [32]. Leaf dry mass (0.25 g_DM_) was extracted with 20 mL of distilled water with 70 % methanol. The mixture was left for 1 h under constant shaking at ambient temperature and light cover. Afterwards, the extract was filtered on Whatman 90 mm paper filter. 1650 µl of distilled water were added to 50 µl of the extract. 200 µl of saturated Na_2_CO_3_ aqueous solution and 100 µl of Folin-Ciocalteu’s reagent were added. After 30 min, the reaction was completed and the extract was measured at 765 nm on a Biomate 3 spectrophotometer (Thermo Electron Scientific Corporation). The quantitative values were expressed in equivalents of gallic acid per leaf dry mass.

### Plant Growth

Growth of the non-destructive set of saplings was measured at the end of the study period (i.e. November) in terms of plant height, basal diameter, fresh and dry leaf mass, shoot and roots dry mass, root/shoot ratio and main root diameter.

### Statistics

Shifts in isoprene emission, gas exchange and chlorophyll fluorescence under the three watering treatments applied over 8 months were tested using a two-way repeated measures ANOVA with treatment (*C*, *MS* and *SS*) and month as factors and the studied traits as dependent variables. Changes in soil and plant water status, LMA as well as concentration of phenolic compounds were analysed using two-way ANOVA, since these parameters were measured on the destructive set of plants. In either case, when these two factors interacted significantly (*P*<0.05), one-way ANOVA was applied for the significant factor. Over the text, simple or repeated ANOVA measurements are named as ANOVA (F). Tukey post hoc tests were used to test differences between months and treatment. Data were log transformed when necessary to achieve normal distribution and homoscedasticity requirements. A Principal Components Analysis (PCA) including the total set of plant traits (growth, emission or concentration of secondary metabolites, physiological and functional traits) in November was performed in order to analyse the adaptation of *Q. pubescens* saplings to the different watering treatments (*C*, *MS* and *SS)*. Linear and non-linear regression analyses were used to evaluate isoprene emission dependency on photosynthesis according to water stress level. Differences between the theoretical and the experimental isoprene emission factors of the same sapling were tested for the three watering treatments (*C*, *MS* and *SS*) using paired sample comparisons (t). Statistical analyses were conducted using R 3.0.3 and Stagraphics software.

## Results

### Soil And Leaf Water Status Under Three Different Watering Treatments

Severe water stress significantly lowered *SRWC* compared to *C* all over the growing season (Table 1, *F_treatment_* = 50.5, *P*<0.001). Mild water stress reduced *SRWC* excepting in fall (September), when it was similar to *C* pots (One-way ANOVA, *F_treatment_* = 3.82, *P* = 0.054, Table 1). Despite the soil dryness caused by water shortage, *MS* never lead to a significant decline of leaf water status (no significant differences occurred in either *LRWC* or *Ψ*_mid_). Only *SS* tended to decrease *LRWC* (at 90% confidence level; two ways ANOVA, *F_treatment_* = 3.2, *P* = 0.047), but their *Ψ*_mid_ only significantly dropped in May and November.

**Table 1:** Indicators of water stress degree.

Parameter	Treatment	April	May	June	July	September	November	Annual mean	Statistics
SRWC(cm^3^.kg^−1^)	C	180.9^a^±21.4	205.9^a^±13.9	226.2^a^±10.8	202.8^a^±7.8	192.0^a^±10.0	222.0^a^±12.5	205.0±7.1	F_treatment_: 50.5; df = 2; *P*<0.001F_month_: 1.1; df = 5; N.S.F_interaction_: 6.0; df = 10; *P*<0.001
	MS	174.6^a^±15.9	144.9^b^±15.4	113.7^b^±13.9	143.5^b^±10.9	200.3^a^±13.7	199.5^a^±12.4	162.8±14.1	
	SS	157.5^a^±13.6	128.2^b^±4.4	135.6^b^±7.3	131.8^b^±7.8	140.7^(b)^±23.4	74.3^b^±11.9	128.1±11.5	
LRWC(%)	C	71.2±3.2	74.1±3.4	78.4±2.0	78.6±3.5	81.0±2.3	85.0±3.1	78.1^A^±2.0	F_treatment_: 3.2; df = 2; *P* = 0.0469F_month_: 6.1; df = 5; *P* = 0.001F_interaction_: 0.7; df = 10; N.S.
	MS	69.9±4.9	71.6±2.0	76.3±2.3	84.9±1.3	82.3±1.6	88.0±1.5	78.8^A^±3.0	
	SS	66.7±5.1	55.5±11.9	69.6±9.8	80.4±1.2	82.3±1.2	78.8±4.1	72.2^(B)^±4.2	
*ψ*_mid_[MPa]	C	1.6^a^±0.2	2.2^a^±0.4	2.3^a^±0.3	2.3^a^±0.4	1.2^a^±0.1	1.0^a^±0.1	1.8±0.2	F_treatment_: 13.1; df = 2, *P*<0.001F_month_: 14.7; df = 5; *P*<0.001F_interaction_: 6.8;df = 10; *P*<0.001
	MS	1.8^a^±0.4	2.5^ab^±0.3	2.8^a^±0.2	2.9^a^±0.3	1.2^a^±0.1	1.2^a^±0.1	2.1±0.3	
	SS	1.8^a^±0.3	4.8^b^±0.8	2.1^a^±0.1	2.4^a^±0.2	1.3^a^±0.1	3.7^b^±0.4	2.7±0.5	
Climaticparameters	T (°C)^(1)^	18.7±0.5	23.3±0.3	23.3±0.2	25.2±0.2	24.4±0.2	17.7±0.1		
	RH (%)^(2)^	63.7±1.5	51.1±0.6	57.8±0.6	52.2±0.5	58.0±0.5	62.0±0.2		

T: mean temperature recorded inside the greenhouses every month; ^(2)^ RH: air relative humidity recorded inside the greenhouses every month.

Soil relative water content (SRWC), leaf relative water content (LRWC) and midday water potential (ψ_mid_) under control, mild and severe water stress (C, MS and SS) all over the growing season. Climatic conditions under the greenhouse are also shown. Variability of these parameters due to water stress, month and their interaction are tested using two-way ANOVA (F) followed by Tukey test. Lower case letters (a>b) represent differences between treatments month by month. Capital letters (A>B) represent differences between treatments all months averaged (Annual mean column). Values are mean ± SE of n = 5; N.S.: not significant. Lower case or capital letters under brackets denote Tukey test differences at 90% confidence level; df: degrees of freedom.

### Physiological Performances (gas Exchange And Fluorescence) Under Water Stress

**Figure 1:**
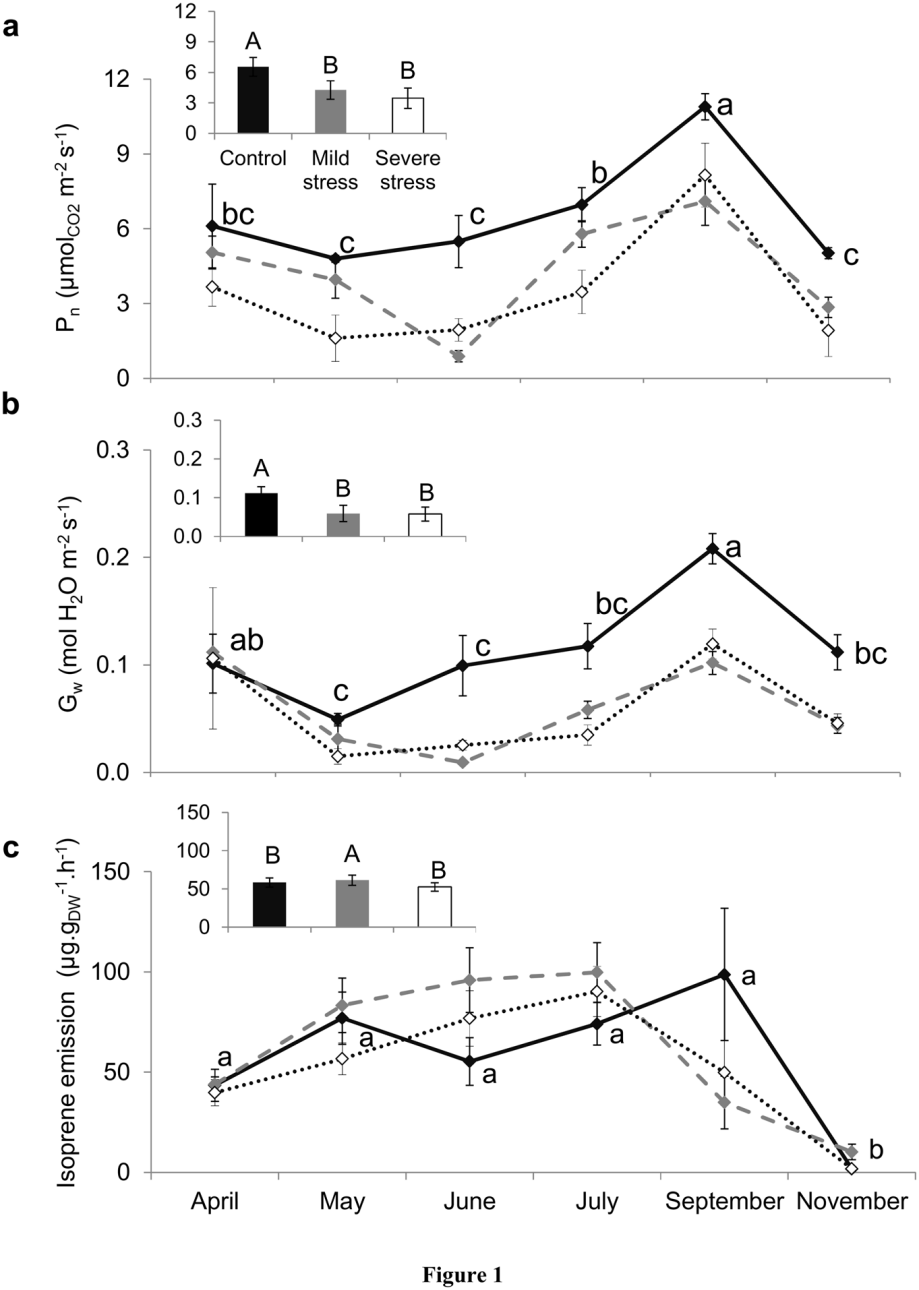
Gas exchanges (photosynthesis, stomatal conductance and isoprene). Seasonal course of the net photosynthesis (P_n_, graph a), stomatal conductance to water vapor (G_w_, graph b) and isoprene emissions (graph c) under control (–⧫–), mild (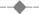) and severe (···⋄···) water stressed. Note that 70% of isoprene emissions were mostly measured under standard conditions (refer to materials and method for details). Differences are tested using a two-way ANOVA repeated measures (F) followed by Tukey tests. Since interaction between seasonality and treatment is not significant according to the two-way ANOVA, results of water stress impact are shown in the small graph where data of all months are pooled together and differences between treatments are denoted by capital letters (A>B) while seasonality impact is shown in the main graph (in lower case letters: a>b>c). Values are mean ± SE of n = 5.

**Figure 2:**
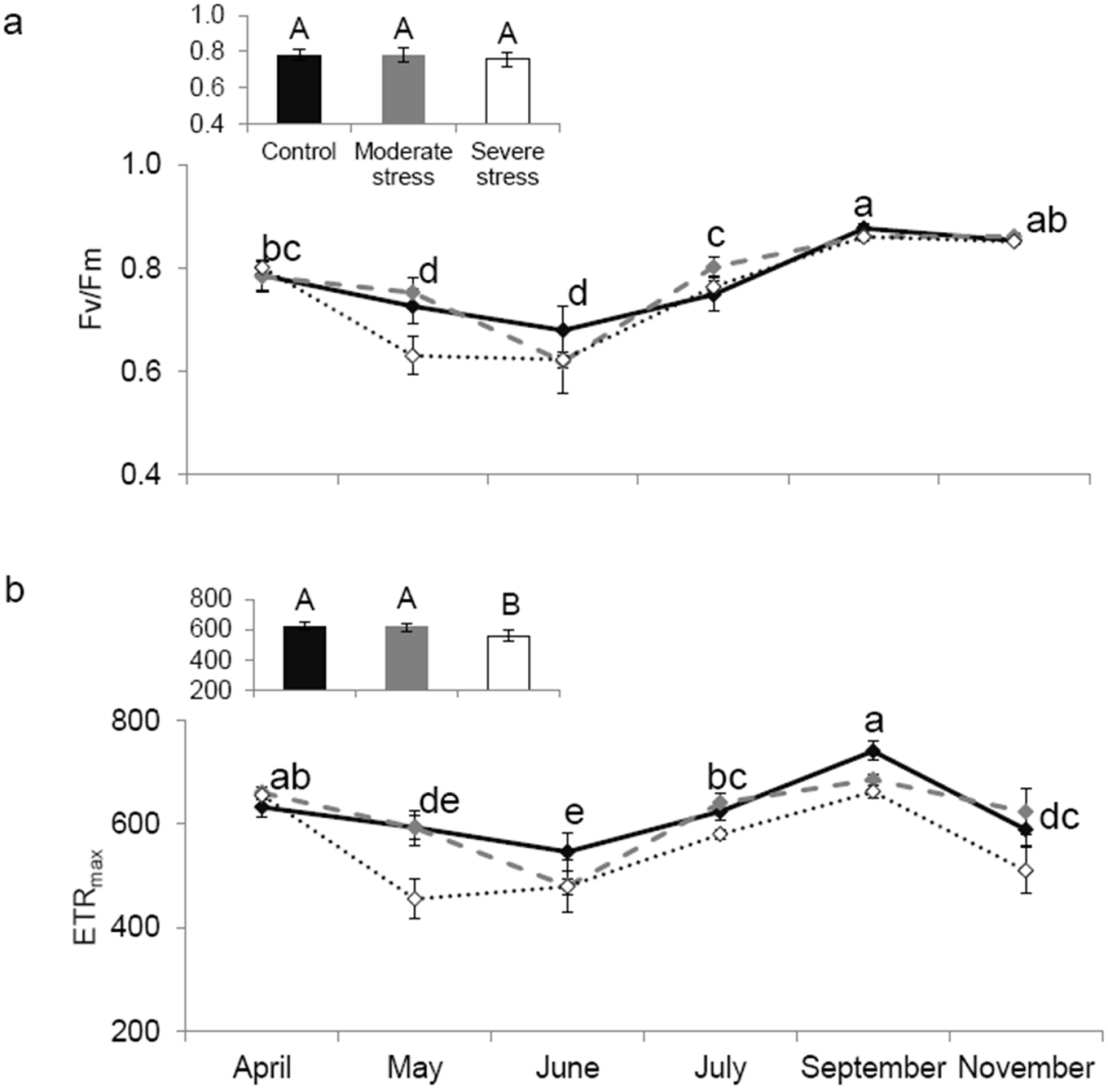
Chlorophyll fluorescence. Seasonal course of the variable to maximum fluorescence ratio (Fv/Fm, graph a) and maximal electron transport rate (ETR_max_, graph b) under control (–⧫–), mild (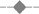) and severe (···⋄···) water stress. Differences are tested using a two-way ANOVA repeated measures (F) followed by Tukey tests. Since interaction between seasonality and treatment is not significant according to the two-way ANOVA, results of water stress impact are shown in the small embedded graph where data of all months are pooled together and differences between treatments are denoted by capital letters (A>B) (similar capital letters indicate the absence of water stress influence) while seasonality impact is shown in the main graph (in lower case letters: a>b>c >d>e). Values are mean ± SE of n = 5.

All over the growing season both, saplings under *MS* and *SS* showed a very significant decline of 35% and 47% on average of *P_n_* and 47% and 48% for *Gw* compared to *C* respectively (Fig. 1a,b, two-way ANOVA, 18.8<*F_treatment_*<23.7, *P*<0.001). *Fv/Fm* did not significantly vary between treatments at any month, while *ETR_max_* was significantly lowered under *SS*, indicating that photosystem II was only damaged under this level of stress (Fig. 2a: *Fv/Fm* two-way ANOVA, *F_treatmen_*_t_ = 1.58, *P* = 0.2131; Fig. 2b: *ETR_max_*_,_ two-way ANOVA, *F_treatment_* = 8.23, *P* = 0.0007).

Physiological status of stressed and non-stressed oaks featured the same seasonal trend with the lowest values of *P_n,_ G_w_, Fv/Fm* and *ETR_max_* in May (one month after water stress was applied) and June, and the highest values of these traits in September, before a decline occurred during leaf senescence in November (two-way ANOVA, 16.0<*F_month_<*31.0, *P*<0.001). Since all physiological traits recovered in September, water stress applied in this study did not cause any irreversible damage. All these physiological traits showed a non-significant interaction between month and treatment (1.05<*F_interaction_*<1.46, 0.178<*P*<0.417, Fig. 1 & 2).

### Isoprene Emissions Under Water Stress

**Figure 3:**
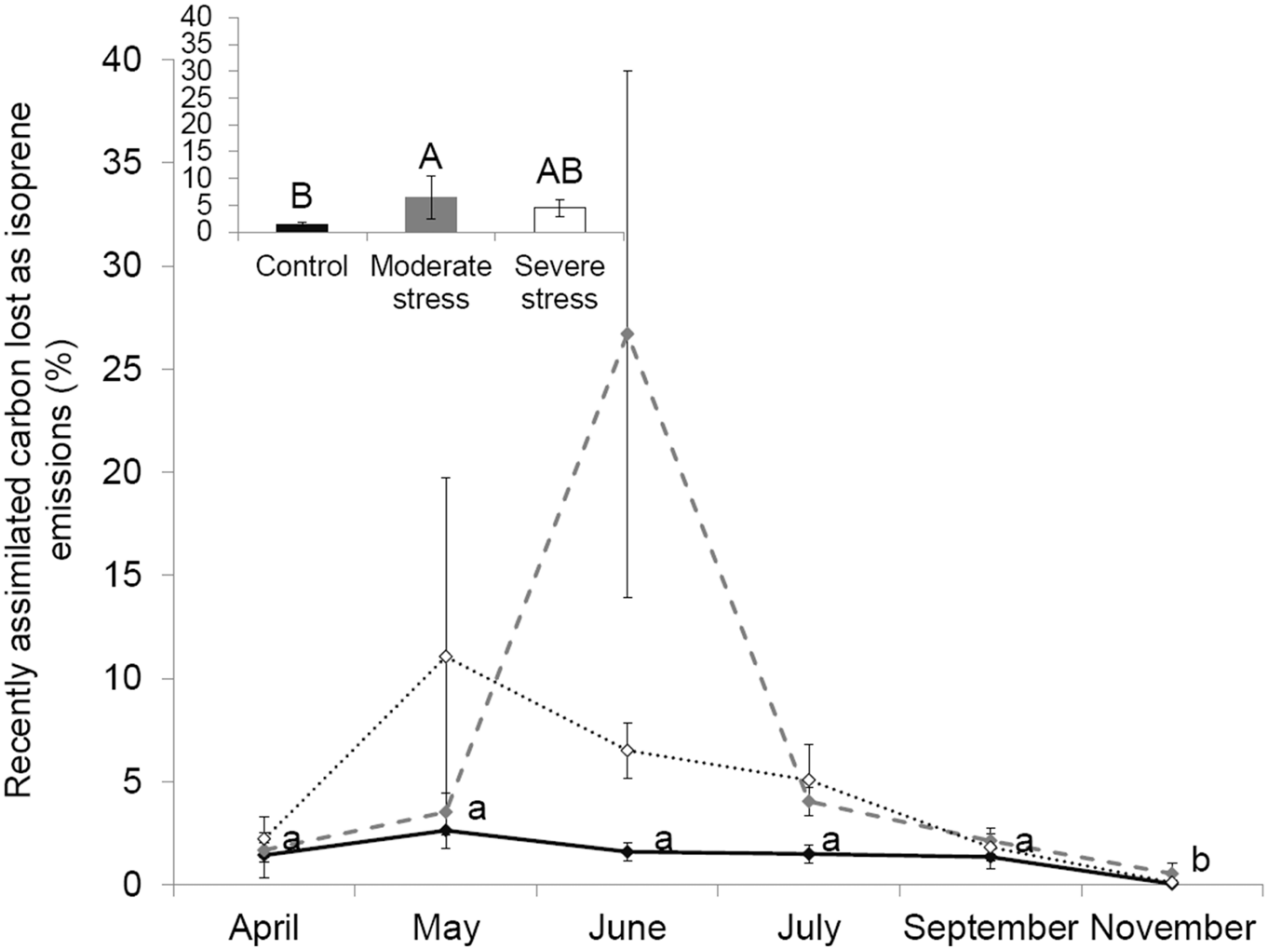
Isoprene emission rate as percentage of carbon re-emitted. Seasonal course of the percentage of photosynthetically assimilated carbon re-emitted as isoprene under control (–⧫–), mild (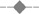) and severe (···⋄···) water stress. Differences are tested using a two-way ANOVA (F) repeated measurements followed by Tukey tests. Since interaction between seasonality and treatment is not significant according to the two-way ANOVA, results of water stress impact are shown in the small embedded graph where data of all months are pooled together and differences between treatments are denoted by capital letters (A>B) while seasonality impact is shown in the main graph (in lower case letters: a>b). Values are mean ± SE of n = 5 saplings.

Mean isoprene emissions under *MS* were statistically higher compared to *C,* while not significant increases occurred in *SS* sapling (Fig. 1c, two-way ANOVA, *F_treatment_* = 4.0, *P* = 0.02; *F_interaction_* = 1.45, *P* = 0.417). Isoprene emission factors showed a similar trend, although emission rates were slightly higher under *SS* (Fig. S1). In June, saplings under *MS* allocated 27% of the photosynthetically assimilated carbon for isoprene emission - the highest fraction attained over the growing season - while it was only 2% in *C* saplings (Fig. 3). In May, in spite of the very restricted *P_n_* featured by *SS* oaks, isoprene emissions remained similar to *C* saplings. As a result, in May, isoprene emissions under *SS* represented 11% of the photosynthetically assimilated carbon (the highest of all the growing season) while it was only 3% in *C* saplings (Fig. 3).

Isoprene emissions (Fig. 1), as well as isoprene emission factors (Figure S1) remained similar from April to September (70.8±5.1 µg g_DM_^−1 ^h^−1^) and declined drastically in November (5.9±3.4 µg g_DM_^−1 ^h^−1^) during leaf senescence. This trend was similar among the three water treatments (two-way ANOVA, *F_month_* = 17.0, *P*<0.001; *F_interaction_* = 1.45, *P* = 0.417).

**Figure 4:**
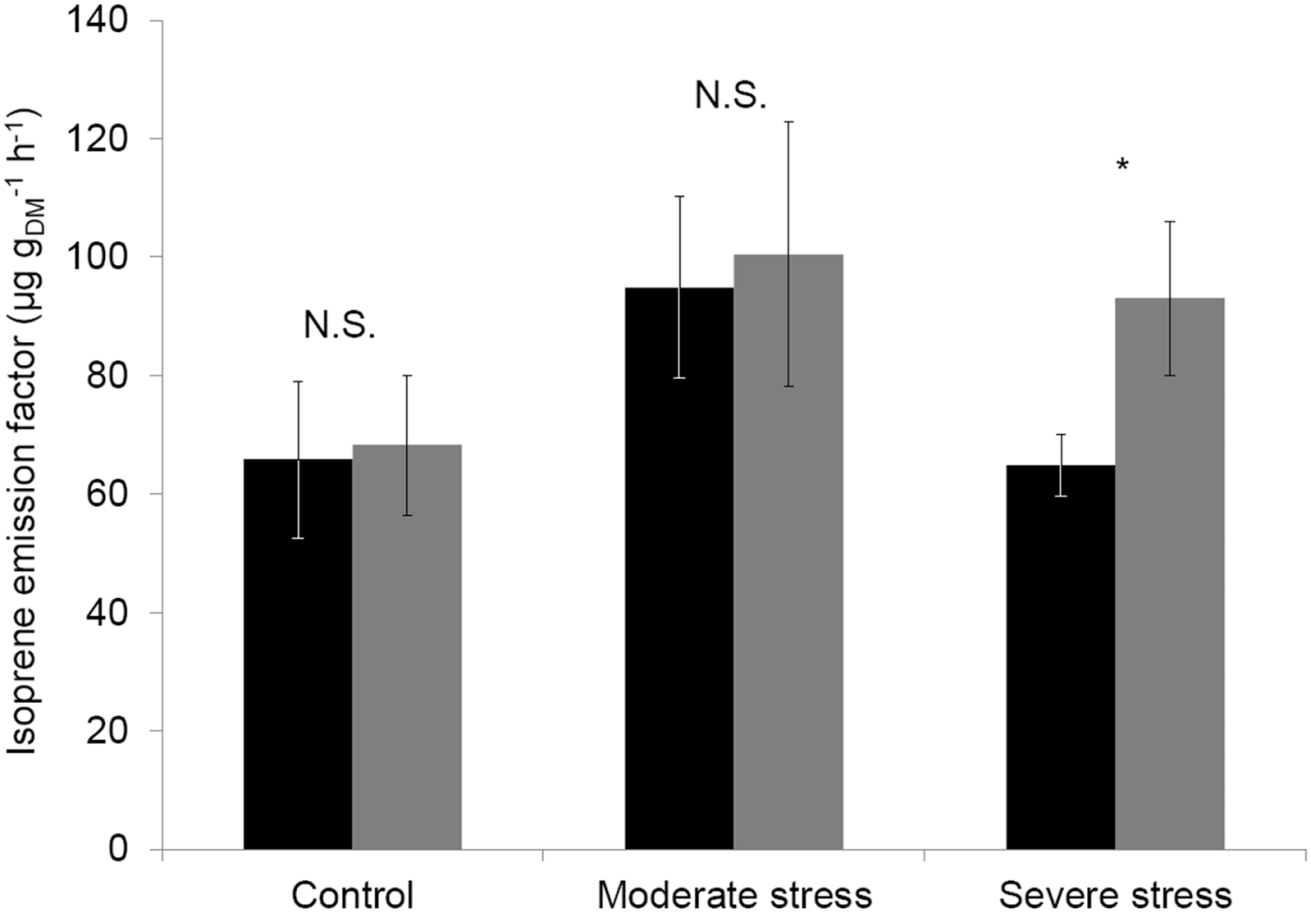
Experimental and calculated isoprene emission factors. Comparison between the isoprene emission factor calculated with Guenther et al. (1993) algorithm (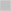) and the experimental isoprene emission factor (▪) obtained under standard conditions (30±1°C and 1000±50 µmol m^−2 ^s^−1^ of PPFD) for control, mild and severely water stressed sapling. t: value of the paired sample comparison tests. N.S.: not significant, *0.01<*P*<0.05. Values are mean ± SE of n = 6–7 saplings.

Isoprene emission rates (ER) were strongly linearly correlated to *P_n_* exclusively in *C* saplings (ER = 5.6+8.0×P_n_, r^2^ = 18.5, *F* = 19.13, *P*<0.001). This correlation was weak although significant in *MS* (ER = 52.1+0.5×*P_n_* ^2^, r^2^ = 6.1, *F* = 3.84, *P* = 0.05) and was not significant under *SS* (*F* = 0.48, *P* = 0.49). The comparison between the theoretical isoprene emission factor obtained using G93, and the experimental isoprene emission factor of the same trees under the three watering treatments revealed that there were no significant differences between *C* and *MS*, while the G93 algorithm overestimated the isoprene emission factor by 44% under *SS* compared to experimental measurements (Fig. 4, paired sample comparisons, *t* = −2.8, *P* = 0.028).

### Concentration Of Phenolic Compounds Under Water Stress

**Figure 5:**
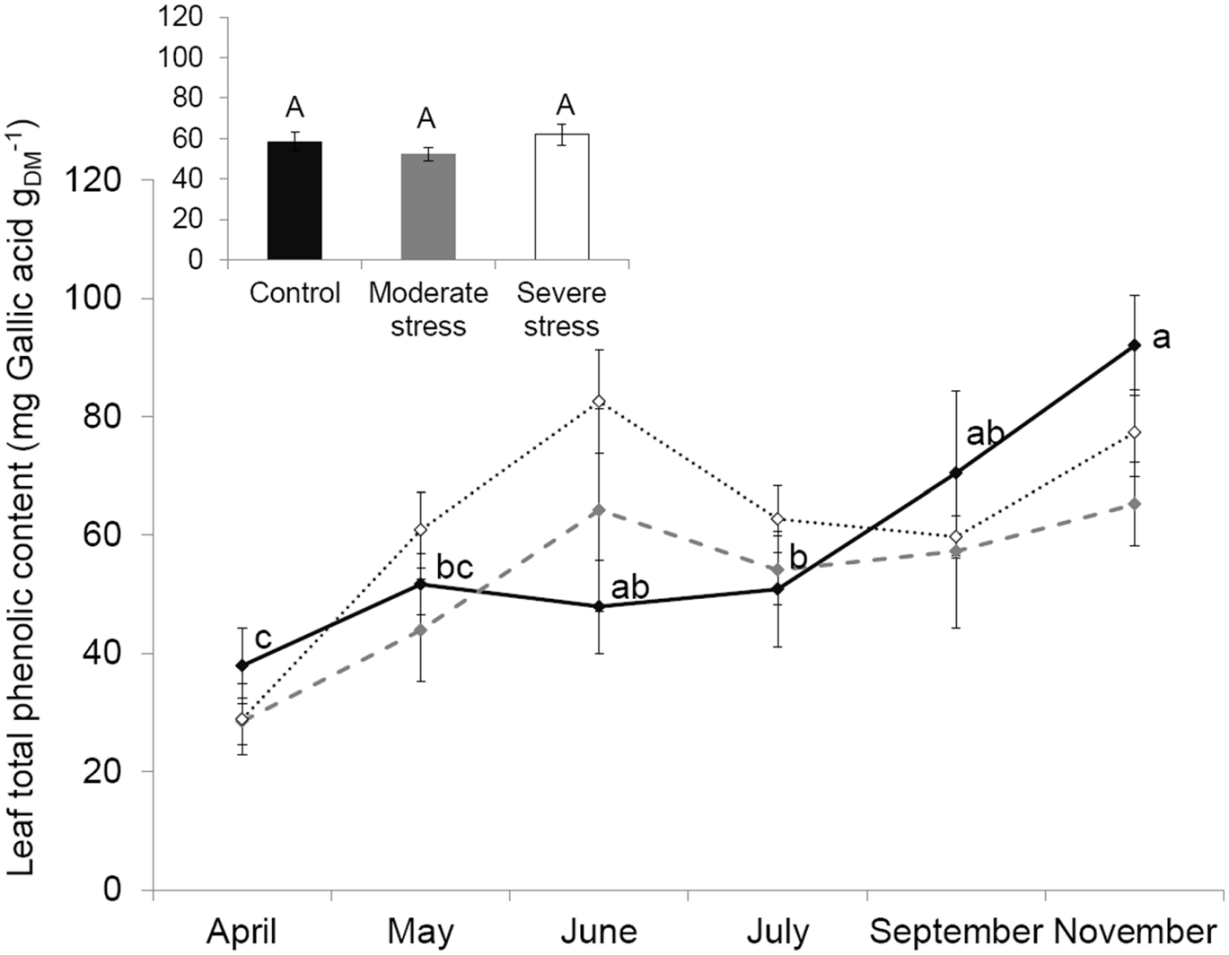
Leaf total phenolic concentration. Seasonal course of leaf total phenolic concentration under control (–⧫–), mild (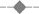) and severe (···⋄···) water stress. Differences are tested using a two-way ANOVA (F) followed by Tukey tests. Since interaction between seasonality and treatment was not significant, the impact of water stress is shown in the small embedded graph where data of all months are pooled together and differences between treatments are denoted by capital letters (similar capital letters indicate the absence of water stress influence) while seasonality impact is shown in the main graph (in lower case letters: a>b>c). Values are mean ± SE of n = 5.

Unlike isoprene emissions, concentrations of phenolic compounds were not statistically sensitive to water stress (two-way ANOVA; *F_treatment_* = 1.38, *P* = 0.26, Fig. 5), independently on the month (*F_interaction_* = 1.34, *P* = 0.23). Their seasonal trend was also different compared to isoprene: *Q. pubescens* produced the lowest phenolic concentrations in April and they increased progressively month by month up to reach the highest values in November (two-way ANOVA, *F_month_* = 8.8, *P*<0.001, Fig. 5).

### Plant Growth Under Water Stress

**Figure 6:**
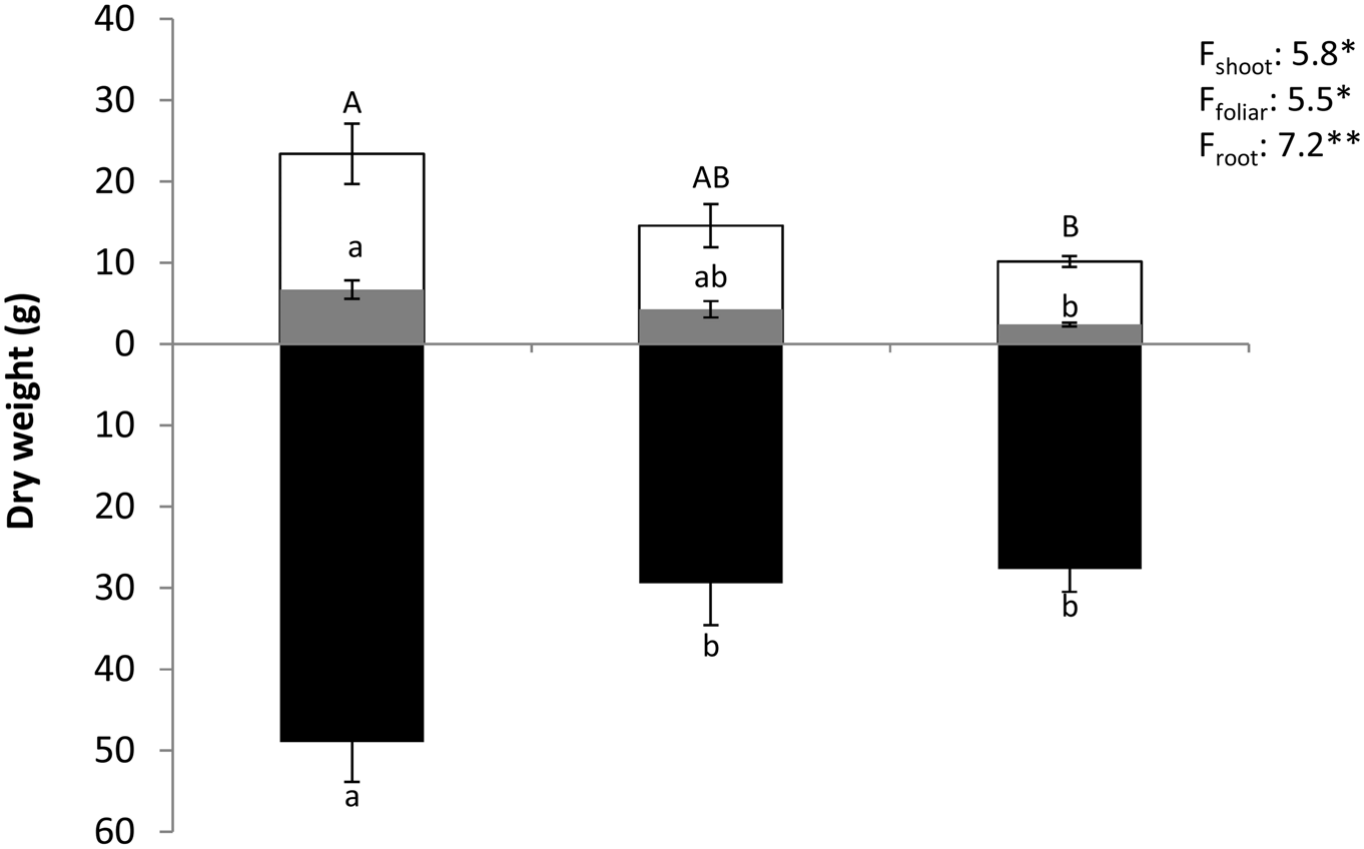
Biomass growth. Shoot, foliar and root biomass (g_DM_ ind^−1^) during leaf senescence (in November) for control, mild and severe water stress. Differences are tested with one-way ANOVA (F). Capital black lower case black and white lower case letters denote statistical differences for shoot, foliar and root biomasses respectively with a>b or A>B given by Tukey tests. Values are mean ± SE. n = 5.

Water scarcity significantly reduced *Q. pubescens* growth. Severely stressed saplings showed a very significant decrease of foliar, shoot and root biomasses and an increase of the root/shoot ratio compared to *C* (one-way ANOVA, 5.8<*F_treatment_*<7.2, 0.008<*P*<0.0182, Fig. 6). Under *MS* conditions, only root biomass was significantly reduced (40% lower than *C*), while shoot and foliar biomasses presented intermediate values between *C* and *SS* treatments.

### Leaf Mass To Area Ratio Under Water Stress

**Figure 7:**
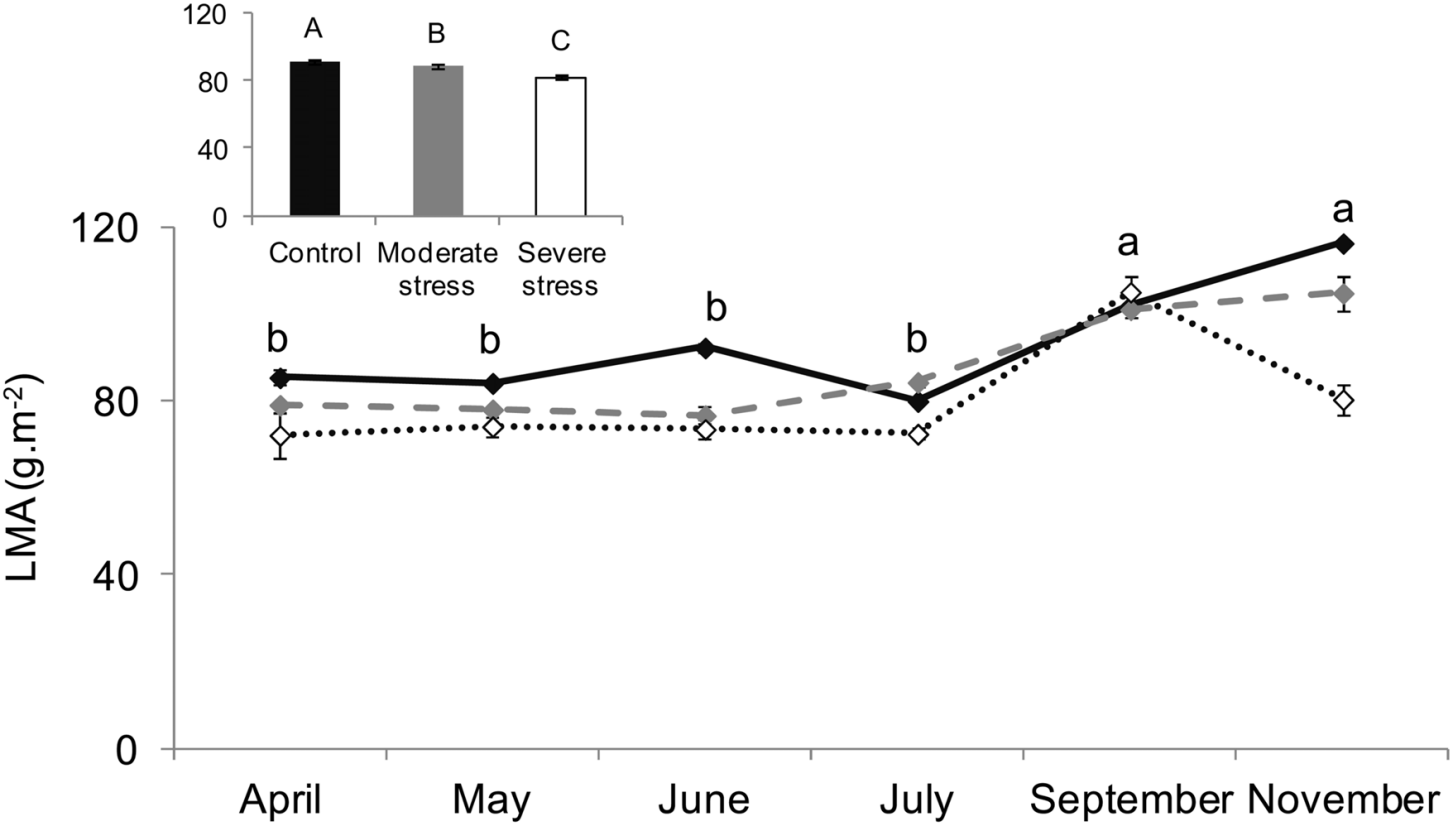
Mapping control, mild stress and severe stress. Two-dimensional mapping of the Principal Component Analysis (PCA) performed for all plant traits measured at the end of the experiment (plant growth, emission or concentration of secondary metabolites, gas exchange and water status). This analysis was performed on n = 5 trees per treatment. Traits shown at the end of some arrows correspond to the most explanatory traits.

LMA was significantly reduced by 7% and 14% under *MS* and *SS* all over the growing season (two-way ANOVA, *F_treatment_* = 7.9, *P*<0.0001, Fig. 7). It also changed over the different months, with the highest values shown at the end of the growing season (November under *MS* and *C* and September under *SS*) and the lowest values from April to July (two-way ANOVA, *F_month_* = 13.7, *P*<0.0001, F_interaction_ = 1.93, P = 0.05).

### Secondary Metabolism, Growth, Physiological And Functional Traits Under Water Stress

**Figure 8:**
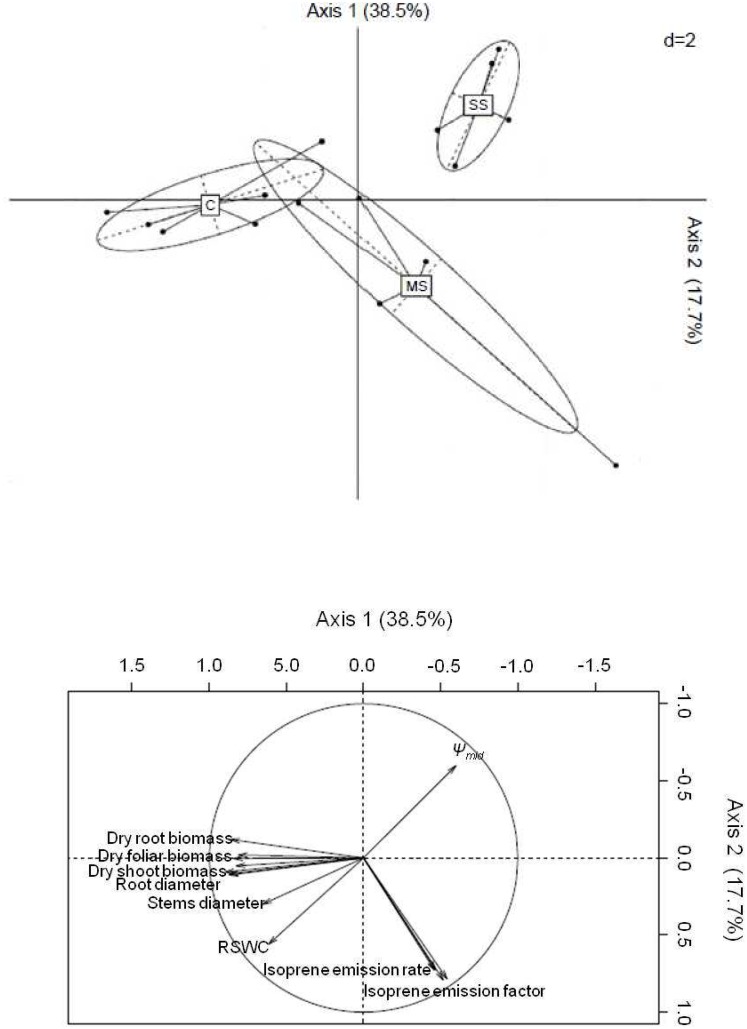
Leaf mass per area ratio. Seasonal course of the leaf mass per area ratio (LMA) under control (–⧫–), mild (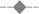) and severe (···⋄···) water stress. Differences are tested using a two-way ANOVA (F) repeated measurements followed by Tukey tests. Since interaction between seasonality and treatment is not significant according to the two-way ANOVA, results of water stress impact are shown in the small embedded graph where data of all months are pooled together and differences between treatments are denoted by capital letters (A>B) while seasonality impact is shown in the main graph (in lower case letters: a>b). Values are mean ± SE of n = 5 saplings.

The PCA - performed with all measured traits in November - revealed that growth, especially root, shoot and foliar biomass, explains sapling repartition on axis 1, while isoprene emissions, followed by Ψ_mid_, explain sapling reparation on axis 2 (Fig. 8). Control saplings featured a high investment in growth biomass. Only under *MS*, saplings were characterized by high isoprene emissions and their growth was only limited in some of them. Under *SS*, saplings were distinguished by a particularly high |***ψ*** *_mid_* | and low growth showing that *Q. pubescens* was unable to maintain the leaf water level and growth rates under such degree of stress. Furthermore, under *SS,* saplings showed a poorer plasticity than *C* and *MS* as reflected by the tight distribution of saplings along both axes.

## Discussion

The significant reduction of *Gw* under water stress impeded a significant water status drop under *MS* as evidenced by the maintenance of LRWC and *Ψ*_mid_. Nardini and Pitt (1999) [33] showed that *Q. pubescens* is capable to preserve high levels of LRWC under drought due to the high efficiency of its water conduction system through the root system. These responses indicate that *Q. pubescens* adaptation to water scarcity can be attributed to a drought-resistant strategy and, more particularly, to a drought-avoidance strategy, known to confer *Q. pubescens* resistance to arid and semi-arid environments. Thus, there were no irreversible leaf damages (*Pn* and *ETR_max_* showed the highest rates in September after the intense summer drought) and photochemical efficiency (*Fv/Fm*) remained unchanged under *C*, *MS* and *SS* all over the study.

Maintenance of similar leaf water levels, especially during *MS*, probably explains why, under this level of water stress, there was a moderate reduction of leaf and shoot growth at the end of the growing season (i.e. at leaf senescence), while there was a marked reduction of root biomass. The ecological advantage of maintaining similar leaf water levels is a low decrease of the leaf area and hence a low drop of CO_2_ uptake before leaf senescence [34]. Additionally, this would confer *Q. pubescens,* a deciduous species, a competitive advantage over the evergreen Mediterranean tolerant species (e.g. *Q. ilex*) during the extended dry periods in summer [35],[36].

Our study also showed that LMA increased at the end of the seasonal cycle and decreased with water scarcity as shown for *Q. ilex* [37], indicating that *Q. pubescens* sclerophylly is not strengthened under the drier conditions applied. Indeed, increases of LMA under water scarcity are associated to the degree of sclerophylly (hardness and thickness) [30] known to promote drought resistance ([37]and citations cited therein). The low LMA values observed under *MS* and *SS* conditions can be explained if the plant prioritizes the allocation of the recently assimilated CO_2_ to other processes different from growth production, such as isoprene synthesis and so isoprene emissions, rather than to produce leaf biomass.

Unlike all these physiological and functional traits, phenolic compounds as a whole did not respond to water scarcity as already reported for *Capsicum annuum* L. [38]. Phenolic compounds are however a very heterogeneous group of secondary metabolites and, as a result, it is likely that the response of different phenolics (e.g. flavonoids, flavonols, anthocyanins, single phenols) to water scarcity varies.

Nevertheless, our study indicates that isoprene emissions were very sensitive to water stress. Isoprene emissions of *C* saplings – around 63 µg g_DM_^−1 ^h^−1^ (or 22 nmol m^−2 ^s^−1^) during most part of the growing season (from April to September) – was indeed significantly increased by 38% on average under *MS* independently on the month, while *SS* caused not significant increases. These results are partly consistent with Sharkey and Loreto (1993) [22] who observed a slight increase of isoprene emitted by *Pueraria montana* under *MS*, even if leaf to leaf variation prevented any statistical significance. Funk et al. (2004) [23] described a similar increase of isoprene emission in *Populus deltoides*.

On the contrary, other studies have not reported isoprene emission shifts during water stress [24],[25] and previous studies on *Q. pubescens* show a decrease in isoprene emissions under *SS* [18],[20]. Differences probably rely on the water stress protocols used. In our study, water stress saplings daily received a lower water supply than *C* saplings over the entire growing season, while the other two cited studies stopped watering in stress sapling while continued watering control saplings, and observed a decrease of isoprene emissions after a period of several weeks of water withholding.

The increase and stability of isoprene under *MS* and *SS* respectively, despite the low *Pn*, reflected an uncoupling between isoprene emissions and *Pn* in agreement with previous investigations [18],[19],[22],[24],[29],[39]. In our study, this uncoupling was especially clear under *SS* since no correlation was found between both processes and isoprene emissions persisted all over the growing season. In the cited studies, the length of time while isoprene emission persisted varied according to the species studied and the degree of stress. It can be also noted that this length is not always mentioned, as most of the studies stop water stress when physiological processes (e.g. *Pn*, *Gw*) reach their minima. As a result, in our study, as much as 3% of the photosynthetically assimilated carbon in *C* saplings was allocated for isoprene emissions in agreement with pas studies [23] and citations therein). By contrast, this percentage was as high as 27% and 11% under *MS* and *SS* respectively. Some authors show that under extreme water limitation, when *Pn* is highly depressed, isoprene emission represents between 20 and 50% of the recently assimilated carbon from photosynthesis [22],[39]. These results suggest that drought resistance *-* in addition to be conferred by stomatal closure before water potential drops as previously described - is probably implemented by increasing the allocation of the recently assimilated carbon to isoprene emissions in view of the benefits isoprene confers for the plants in terms of cell oxidation reduction and cell stabilization [3],[9].

Niinemets (2010) [15] proposed that mild stress decreases stomata openness and therefore, carbon assimilation, but does not inhibit isoprene emissions since sugars, starch reduction, and *ETR* are maintained. This later hypothesis was confirmed in our study where *ETR_max_* values under *C* and *MS* conditions were similar. Other authors claim that other carbon sources stored in the plant, rather than recently assimilated carbon, become available to support isoprene biosynthesis under stress conditions [23]. These alternative sources seem hence to be extra-chloroplastic, such as xylem-transported glucose in *Quercus robur* coming from root and stem storage [23],[24],[40]. In view of the previous results, Rodríguez-Calcerrada et al., (2012) tested if carbohydrate accumulation in leaf cells of *Q. pubescens* were promoted and used for isoprene biosynthesis under water stress. Although authors reported an increase in soluble sugars under water stress, a decrease in isoprene emission was observed. They deduced that water stress reduced the availability of phosphoenolpyruvate. In our study, *MS* could have favoured phosphoenolpyruvate transportation from cytosol to chloroplasts where isoprene is synthesized [41].

The marked uncoupling between isoprene emissions and *Pn* under *SS* also reflects that isoprene emissions are not well explained by light and temperature under such degree of stress as also shown for other isoprene emitters (*Quercus serrata* Thunb., *Quercus crispula* Blume. [19]). Hence, under *SS* there was a significant difference between the calculated isoprene emission factor using G93, which relies on temperature and light conditions, and the experimental isoprene emission factor. Our results indicate that the traditional G93 algorithm for isoprene standardization, overestimates isoprene emissions by 44% under *SS* conditions.

To conclude, the results obtained are partly in agreement with the GDBH as *MS* promoted isoprene emissions as expected but, it was clearly detrimental for *Pn* and only slightly reduced growth (Fig. S2). The opposite underlying mechanism is however stated by the theory, that is, a clear growth reduction and a slight *Pn* limitation, generating an important pool of carbon which remains available for isoprene emission (Fig. S2). Our study indicates that isoprene emission increase under *MS* is rather due to the rise of the CO_2_ fraction that is re-emitted as isoprene. Under *SS*, isoprene emissions were as low as under *C* conditions and, as stated by the GDBH, this pattern was associated to an important growth reduction and a dramatic decline of *Pn*.

It is likely that increases of isoprene emissions (as well as the isoprene emission factor) under *MS* (up to 27% of the assimilated CO_2_ is reemitted as isoprene) confers a high competitive ability to this species under a climatic scenario moderately dry. Since isoprene emitting biomass (i.e. foliar biomass) was not significantly reduced when *MS* was applied to *Q. pubescens,* it would be interesting to model the consequences on O_3_ and SOA formation, especially in the Mediterranean area, where this oak accounts for the main isoprene- emitting species. By contrast, the marked overall growth reduction observed under *SS*, suggests that investment in isoprene emissions under a very dry scenario would imply a poor fitness of *Q. pubescens.* We could hence expect a lower direct implication of this oak in atmospheric pollution processes compared to a moderately dry scenario, if conditions (e.g. temperature and light) driving the photochemical reactions necessary for SOA and O_3_ formation were similar under mild and severe drought.

We suggest that future investigations explore the competitive advantages of plant investment in isoprene emissions under natural conditions as their adaptive mechanisms to drought could vary compared to young potted oaks, analyse the response of different phenolic groups to drought, and model the formation of biogenic SOA and O_3_ from isoprene under different drought conditions.

## Supporting Information

Figure S1
**Isoprene emission factor.** Isoprene emission factor over the growing season for control (–⧫–), mild (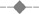) and severe (···⋄···) water stress. Differences are tested using a two-way ANOVA repeated measurements (F) followed by Tukey tests. Since interaction between seasonality and treatment is not significant according to the two-way ANOVA, results of water stress impact are shown in the small graph where data of all months are pooled together and differences between treatments are denoted by capital letters (A>B) while seasonality impact is shown in the main graph (in lower case letters: a>b>c). Values are mean ± SE of n = 5.(DOCX)

Figure S2 **Balance between growth and carbon-based secondary metabolism.** Response of carbon-based secondary constitutive metabolites, growth and net photosynthesis to resource availability as stated by the Growth Differentiation Balance Hypothesis (graph A) and as observed for isoprene emissions in this research study (graph B). (DOCX)
