# Supplementary material for: Isoprene Emissions from Downy Oak under Water Limitation during an Entire Growing Season: What Cost for Growth?
Source: PLoS One. 2014 Nov 10;9(11):e112418. doi: 10.1371/journal.pone.0112418 (PMC4226567; doi:10.1371/journal.pone.0112418)
Supplement: Figure S1 — (DOCX) [file pone.0112418.s001.docx]

**Supporting information**

**Isoprene emissions from Downy Oak under water limitation during an entire growing season: what cost for growth?**

Anne-Cyrielle Genard-Zielinski ^1,2^, Elena Ormeño ^1 (*)^, Christophe Boissard^2^, Catherine Fernandez ^1^

^1^ Institut Méditerranéen de Biodiversité et d’Ecologie marine et continentale (IMBE) Aix Marseille Université, CNRS, IRD, Avignon Université, Technopôle Arbois-Méditerranée. 3 Place Victor Hugo, 13331 Marseille cedex 3, France.

^2^ Laboratoire des Sciences du Climat et de l’Environnement (LSCE-IPSL), Unité Mixte CEA-CNRS-UVSQ (Commissariat à l’Energie Atomique, Centre National de la Recherche Scientifique, Université de Versailles Saint-Quentin-en-Yvelines), F-91198 Gif-sur-Yvette, France.

**Fig S1**
