# Supplementary material for: Isoprene Emissions from Downy Oak under Water Limitation during an Entire Growing Season: What Cost for Growth?
Source: PLoS One. 2014 Nov 10;9(11):e112418. doi: 10.1371/journal.pone.0112418 (PMC4226567; doi:10.1371/journal.pone.0112418)
Supplement: Figure S2 — (DOCX) [file pone.0112418.s002.docx]

**Fig S2.**
